# Analysis of Nonlinear Transient Energy Effect on Thermoelectric Energy Storage Structure

**DOI:** 10.3390/ma13163639

**Published:** 2020-08-17

**Authors:** Jia Yu, Hongji Zhu, Li Kong, Haoqing Wang, Jiawen Su, Qingshan Zhu

**Affiliations:** College of Aerospace and Civil Engineering, Harbin Engineering University, Harbin 150001, China; yujia@habru.edu.cn (J.Y.); kassie@hrbeu.edu.cn (L.K.); S317020097@hrbeu.edu.cn (H.W.); sujw@avic.com (J.S.); zhuqingshan@hrbeu.edu.cn (Q.Z.)

**Keywords:** thermoelectric generator (TEG), phase change materials (PCMs), transient response, optimization

## Abstract

In complex flight conditions, due to the large amount of unusable heat generated by aerodynamic heating, the thermal protection system of an aircraft needs to withstand a large temperature shock, which brings great challenges to the design of the structure. In order to effectively utilize the irregular aerodynamic heat, and improve structural heat conduction, a composite structure is formed by using phase change energy storage materials on the basis of the thermoelectric structure, which transforms the aerodynamic waste heat into stable electric energy for the internal system. Through the study of the response of nonlinear transient energy, it is found that the thermoelectric and mechanical properties of the new structure can be improved by adding phase change energy storage materials. Under actual flight conditions, the new structure can reduce the maximum temperature by 180 K and the maximum thermal stress by 110 Mpa. The mechanical properties of the structure are effectively improved, the service life of the structure is prolonged, and the waste heat can be converted into stable electrical energy output to improve the thermoelectric output performance. On the premise of ensuring conversion efficiency, the output power of the new structure has been improved by 64.8% through structural optimization under actual flight conditions.

## 1. Introduction

With the development of thermoelectric materials (TEMs) [1,2], the thermoelectric generator (TEG) began to be widely used in waste heat recovery [3,4]. The TEG is usually applied to convert thermal energy into electrical energy by exploiting the Seebeck effect [5,6]. This is based on the temperature gradient between the hot and cold sides of thermoelectric elements, which results in heat flux through these elements, a small percentage of which is converted into electrical power [7,8]. It has the unique advantages of high reliability and quiet, environmentally friendly operation [9], and its application field has been widely studied [10,11]. Chen et al. have shown that fluctuating temperature boundary conditions have adverse effects on TEG output performance [12,13]. Zhang et al. found that fluctuating conditions of the hot side of the TE module will lead to instability and low efficiency of the TEG system [14]. Jia et al. analyzed the response under an exponential transient heat source. Therefore, finding a way to stabilize the hot terminal temperature and improve the thermoelectric performance has become the focus of researchers.

Phase change materials (PCMs) can be used in phase change energy storage technology to reconcile the contradiction between heat energy supply and demand in time and space [15,16,17]. PCMs are widely used in industrial waste heat recovery, building energy saving, power peak cutting and valley filling, solar energy utilization, military engineering, aerospace and other fields [18,19]. In addition, PCMs can also be used in the temperature control system because the temperature is approximately constant during the phase transformation [20]. Furthermore, the energy accumulated in PCMs is available in reverse TEG operations, which prolongs the TEG’s work time [21].

In actual flight conditions, the external heat source cannot be constant, which leads to fluctuations in the efficiency and output energy of thermoelectric devices. In addition, excessive stress can reduce the service life of thermoelectric generators [22]. Therefore, more and more researchers have been attracted to the study of the mechanical reliability of structure in recent years [23,24,25]. Forming a stable temperature difference at two ends of the thermoelectric device is one of the main methods of improving the thermoelectric and mechanical properties of the thermoelectric device [26]. However, few studies have attempted to broaden the operating temperature range of thermoelectric devices, especially for energy harvesting from ambient temperature fluctuations.

The actual working condition of a TEG considered in this paper is the aerodynamic heat of aircraft, which has strong fluctuation and which will cause great temperature impact on the TEG, while its performance can be effectively improved under the action of phase change materials. By combining the phase change energy storage structure with the thermoelectric structure, a relatively stable temperature difference is formed at both ends of the thermoelectric device, enabling a study of the response of the whole thermoelectric energy storage structure to the nonlinear transient energy, and an analysis of its thermoelectric and mechanical properties.

## 2. Model Design and Verification

### 2.1. Finite Element Model

**Figure 1:**
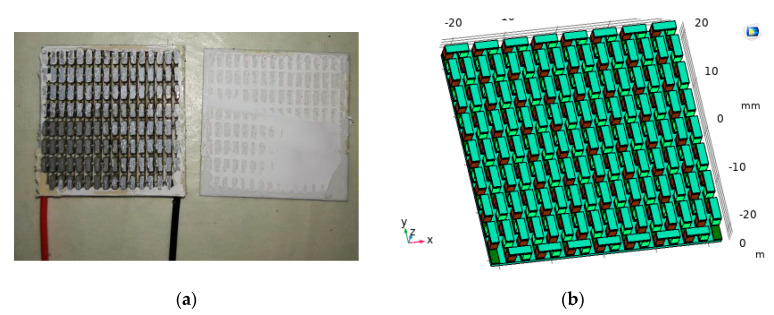
(**a**) Commercial thermoelectric plate; (**b**) COMSOL finite element model.

**Figure 2:**
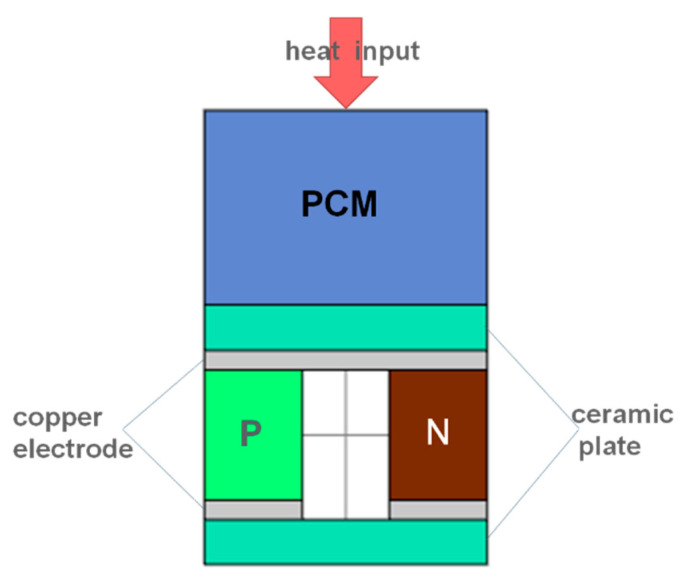
Thermoelectric energy storage structure.

Commercial finite element software COMSOL Multiphysics 5.4 was used for numerical simulation. The three-dimensional finite element model was established with 127 pairs of P-N junction commercial thermoelectric plates as the prototype, as shown in Figure 1. The size of the thermoelectric leg was 1.5 mm × 1.5 mm × 2 mm, the ceramic plate thickness was 0.7 mm, and the copper electrode thickness was 0.3 mm. In order to be as close to the actual material as possible, the Bi_2_Te_3_ was selected as the thermoelectric materials [13]. The composite structure of phase change energy storage structure and thermoelectric structure is shown in Figure 2, with single P-N junction as an example.

### 2.2. Boundary Condition

Some assumptions were made in the simulation, ignoring those factors that had little impact or could not be considered in the model.

(a). Without considering the influence of environmental factors, it was assumed that all surfaces are thermal insulation except the cold and hot ends.
(b). The effect of contact resistance and contact thermal resistance were not considered.
(c). In the experimental verification model, the hot and cold ends were different composite phase change materials, so as to form a temperature difference corresponding to the experiment.
(d). In the transient simulation model, the suitable composite phase change material was selected at the hot end according to the temperature, and the ambient temperature was 293.15 K at the cold end.

### 2.3. Experimental Verification

The composite PCMs with high thermal conductivity and high latent heat were prepared by the melt infiltration method [27]. The porous matrix material with high melting point was used as the skeleton, and the phase change material with low melting point was melted into the porous matrix. According to the actual applicable temperature, two kinds of phase change materials including paraffin and erythritol were selected, and graphite foam was used as the skeleton. The high thermal conductivity graphite foam was dried and placed in a crucible, and the sufficient PCMs were cut into pieces. Then the container was placed in the heating bellows, the temperature was set to 350 K and 420 K for half an hour to ensure that the PCMs had completely melted and infiltrated into the graphite foam skeleton. Finally, the composite PCMs were obtained by removing the impurities on the surface after complete solidification.

Two kinds of composite phase change materials were selected from Table 1, and the phase change temperatures were 324 K and 390 K, respectively.

**Figure 3:**
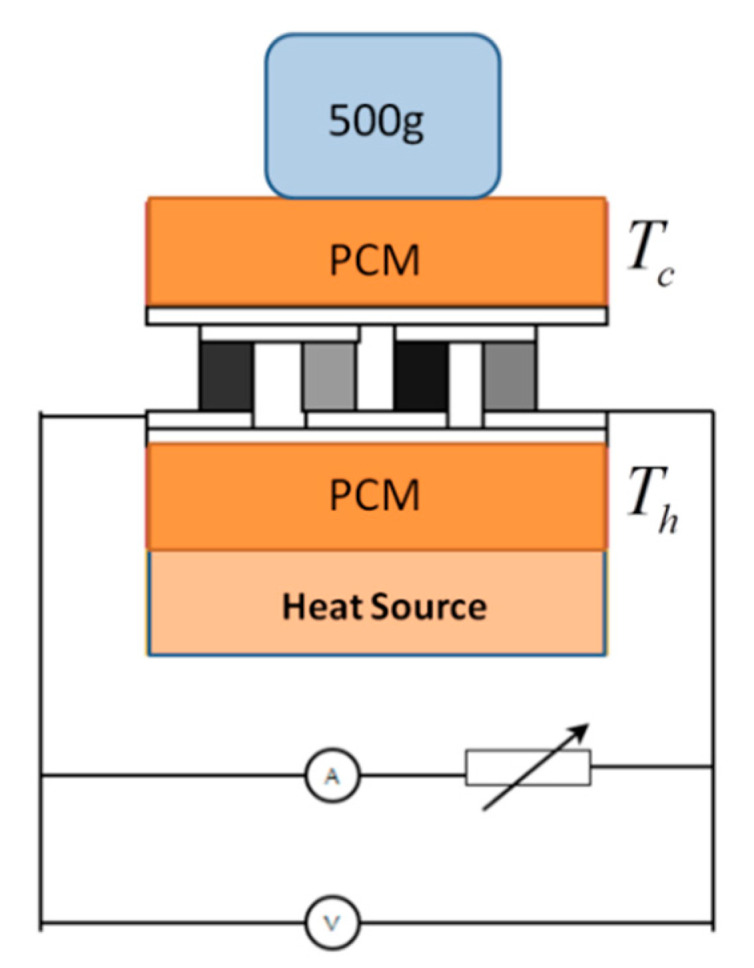
Schematic diagram of experimental device.

**Figure 4:**
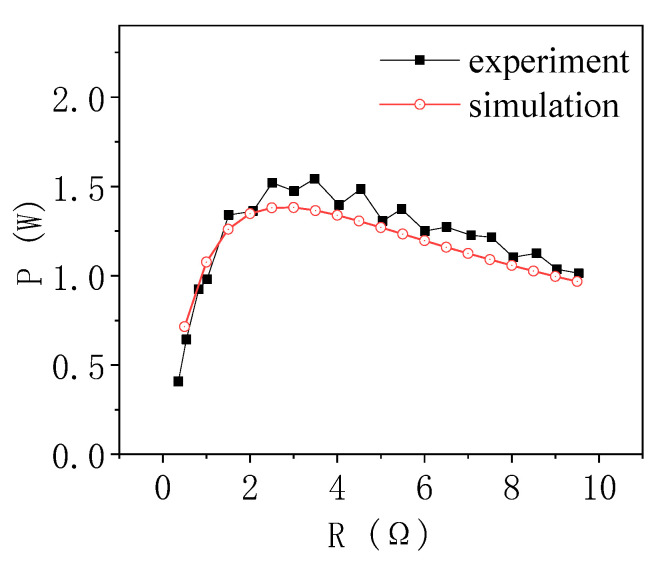
Output power varies with load resistance.

The experiment was carried out on the experimental platform in Figure 3, and phase change materials were also used to control the temperature of the cold end. The composite phase change materials with a hot end and a cold end selected were erythritol and paraffin respectively. Theoretically, a temperature difference of 66 K can be formed after stabilization. The experimental data is shown as the black curve in Figure 4. At the same time, the finite element software was used for simulation, and the data is shown in the red curve in Figure 4. Comparing the simulation results with the experimental results, it was found that the trend of the curve obtained from the simulation and the experiment was basically the same. With the change of the load resistance, the output power had a maximum value. At this time, the corresponding load resistance value should be the internal resistance.

Considering that the temperature difference between the two ends of the TEG composite phase change materials was not necessarily 66 K, the influence of contact resistance and contact thermal resistance was ignored in the model establishment. Since the material parameters of commercial thermoelectric plate were not detailed, the material parameters as close as possible from the literature were selected, which may lead to the difference between the experimental results and the simulation results. Therefore, considering the experimental error and model simplification, it was considered that the simulation was in good agreement with the experiment, and the TEG finite element simulation was effective.

## 3. Results and Discussion

In order to study the response of a thermoelectric energy storage structure to transient energy input and to analyze its thermoelectric and mechanical properties, different forms of heat flow were selected as the input conditions of the hot end to simulate two different working conditions. The first was regular impulse heat flow [28], and the second was irregular heat flow of an actual flight mission [29]. Through the study of the response of the new thermal energy storage structure to the two heat flows, the thermal and mechanical properties of the basic thermoelectric structure and the new structure after adding phase change energy storage materials were compared, reflecting the influence of the phase change energy storage structure on the performance.

The structure connecting a p-type material with an n-type material is called a PN junction. Considering the periodic symmetry of the thermoelectric structure, in order to simplify the model, eight pairs of PN junctions were selected as the reference model for calculation. In order to study the influence of the phase-change energy storage structure on the performance of the hot end, mannitol-graphite foam composite phase change material with phase change temperature of 438 K was selected according to the temperature range. It was considered that the cold end had a good convective heat transfer ability, and the cold end was set as the ambient temperature.

### 3.1. Regular Pulse Heat Flow

**Figure 5:**
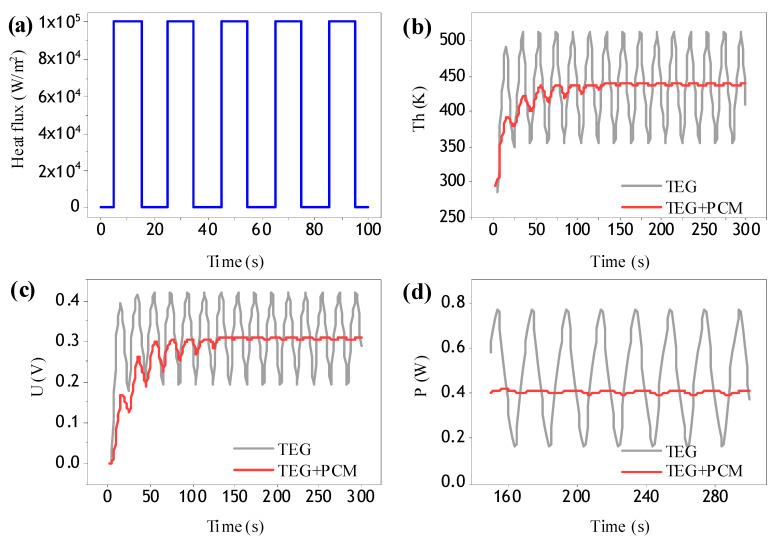
(**a**) Regular pulse heat flow; (**b**) Temperature curve; (**c**) Voltage curve; (**d**) Power curve.

The regular pulse heat flow was applied as shown in Figure 5a to the structure. Comparing the stable state of the temperature curve at the hot end (after 150 s) from Figure 5b, after adding phase change materials to the structure, the hot end temperature was stable near the phase change temperature of the composite phase change materials, which effectively reduced the temperature fluctuation. It is beneficial to protect the thermoelectric structure from damage due to the temperature of the hot end exceeding the service temperature and, furthermore, it is beneficial to reduce the alternating thermal stress and improve the service life and reliability of the thermoelectric structure.

According to the comparison of output voltage and output power curve (after 150 s) in Figure 5c,d, for the basic thermoelectric structures, the output voltage fluctuated greatly (0.2 V~0.4 V) and the output power also fluctuated greatly (0.17 W~0.77 W), so it was difficult to find a suitable service condition in a practical application, and it will also affect the electrical performance of subsequent connected devices. After the addition of the composite phase change material, the voltage was stabilized in a small fluctuation range. Basically, it could be considered that the voltage output with a constant value of 0.3 V could be provided in actual use, and the output power was stable at 0.4 W.

Therefore, from the comparison of the above performances, it can be seen that the use of composite phase change materials plays a positive role in reducing alternating thermal stress and improving the output performance. Therefore, it can be considered that the addition of composite phase change materials is very effective under the relevant boundary conditions in this study. It is found that the output performance curve of a thermoelectric structure is basically the same as the temperature curve of the hot end, that is, the temperature difference between the cold end and hot end plays a decisive role in the output performance.

### 3.2. Actual Flight Heat Flow

**Figure 6:**
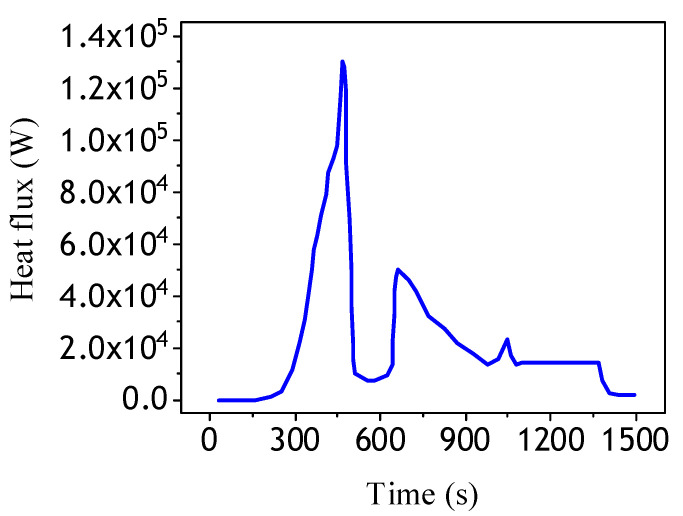
Heat flow of actual flight mission.

For actual flight conditions, aerodynamic heat flow does not have such a strong regularity, so typical aircraft aerodynamic heat flow is selected for the simulation calculation [29], and the input heat flow curve is shown in Figure 6.

**Figure 7:**
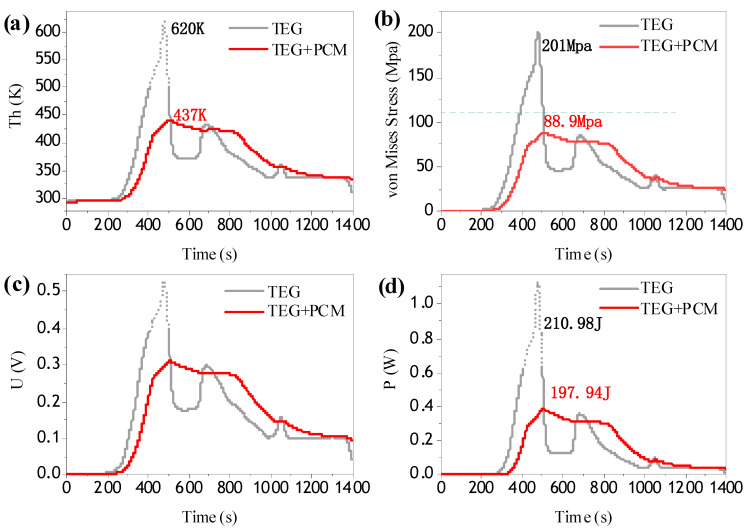
(**a**) Temperature curve; (**b**) Stress curve; (**c**) Voltage curve; (**d**) Power curve.

With the actual flight heat flow in Figure 6 as the input of the hot end, the temperature curve of the hot end in Figure 7a shows that the structure with the addition of the composite phase change material has a good effect on peak and valley elimination:

(1) Without phase change materials, the peak temperature reached 620 K, well beyond the applicable temperature range for thermoelectric materials. Excessive temperature will cause structural damage and performance failure. However, the structure with phase change material reduces the peak temperature by 180 K and is stable within the applicable temperature range of thermoelectric materials, which can effectively protect the thermoelectric structure. It not only prevents the damage to the structure caused by the instantaneous pulse heat, but also expands the applicable temperature range of the composite thermoelectric structure.
(2) Valley removal effectively extends the existence time of stable temperature difference after 500 s, that is, the actual temperature difference generation time is extended, so that the total heat is utilized more effectively.

Due to the good performance of the thermoelectric structure, a large temperature difference is needed on both sides, so the thermal stress generated by the temperature gradient is always an unfavorable factor; too much thermal stress will cause damage to the structure, and reduce the service life [30]. The von Mises stress curve in the thermoelectric leg is shown in Figure 7b. As the phase change material reduces the peak temperature, the thermal stress generated by the temperature difference is also reduced, which effectively relieves the stress inside the structure. According to the material parameters in the references [13], the yield stress of the material selected in this paper is 112 Mpa, and the new structure effectively reduces the maximum thermal stress to the yield limit. It is helpful to reduce the failure probability of the structure and extend its service life. Therefore, the combination of phase change energy storage structure and thermoelectric structure has a very important role in improving the mechanical properties.

As shown in Figure 7c,d, the structure of the phase-change material was used to transform the instantaneous pulse electrical energy output into a stable and lasting electrical energy output. In terms of voltage, a sudden voltage shock may cause damage to subsequent devices connected with the thermoelectric structure. After integrating the output voltage against time, the total output energy changed from 210.98 J to 197.94 J after stabilization, only reducing by 6.2%, which is completely acceptable.

Under the condition of nonlinear transient energy input, the thermoelectric energy storage structure composed of the thermoelectric structure and composite phase-change energy storage material stabilized the temperature of the hot end and converts waste heat into relatively stable electrical energy output, giving full play to the energy storage function of the phase change material and the thermoelectric structure. In the case of regular heat flow, the thermoelectric energy storage structure had a more obvious effect on the stability of electric energy output, which can be regarded as a constant electric energy output. Considering the actual flight conditions, the composite structure can effectively resist the temperature impact and improve the internal stress distribution. Due to the great uncertainty of actual flight conditions, the peak temperature and valley values brought by the aerodynamic heating effect may vary greatly, which may generate great thermal stress. In this case, the PCM effectively reduced the temperature fluctuation. To sum up, adding composite phase change energy storage materials to the thermoelectric structure plays a very positive role in the mechanical and thermoelectric properties of the structure.

### 3.3. Optimization of Actual Flight Heat Flow

By analyzing the temperature curve in the previous section, it was found that the composite phase-change material will stabilize its temperature near the phase-change temperature. Therefore, in this part, the actual flight heat flow will be taken as the working condition, and the temperature boundary conditions will be set to continue to study the output performance of the thermoelectric structure. That means the temperature of two ends are 410 K and 293.15 K respectively. The size of the thermoelectric legs is optimized with the size of the commercial thermoelectric board as the initial value and the structural weight as the optimization goal. A comprehensive consideration of space utilization and thermoelectric performance, under the premise of ensuring a certain conversion efficiency, finds the best value of output performance.

Considering the periodicity and symmetry of the model, in order to reduce the consumption of computing resources, eight pairs of thermoelectric leg models were selected for calculation in the optimization of this section. The overall ceramic plate size was 10.5 mm × 10.5 mm, and the four parameters for optimization were respectively leg side length (L_P, L_N), leg height (H) and load resistance (R). The target was the optimal value of output power (P), and the conversion efficiency should not be too low.

The optimization results are shown in Table 2:

After optimization, the output power was increased from 0.2617 w to 0.4313 w, an increase of 64.8%, and the conversion efficiency of 4.99% was completely acceptable. Here, to introduce the concept of volume power density and per unit volume of the output power, the calculation for the output power and the ratio of total volume, and the value of the size of the main body structure of space utilization high and low, the optimal results can be seen. The power density from the foundation of ascension 5.934 × 10^5^ W/m^3^ to 1.303 × 10^6^ W/m^3^ increased by 119.6%, significantly improving the space utilization and reducing the thermoelectric structure at the same time to guarantee the performance of the total thermoelectric volume and weight.

## 4. Conclusions

In this paper, based on the thermoelectric structure, composite phase change energy storage materials were introduced to form a new thermoelectric energy storage structure, which plays a good role in the mechanical properties and thermoelectric properties of the original structure. It also optimized the thermoelectric structure under actual working conditions, so that the performance was improved.

By comparing the response of the basic thermoelectric structure and the new structure with PCM to the flight heat flow, it was found that the positive effects of introducing phase change energy storage materials are mainly manifested in three aspects:

The peak temperature of the two heat flow conditions was reduced from 513 K and 620 K to the phase transition temperature near 437 K, which effectively alleviated the high temperature shock and protected the internal structure. That means the use temperature of the TEG can be increased by selecting suitable phase change materials.

The maximum thermal stress was reduced by about 110 MPa, which effectively improved the reliability and service life of the structure.

It can improve the thermoelectric output performance and ensure the normal use of subsequent devices by making the large fluctuation electric energy more stable.

The trend of the thermoelectric output performance curve is the same as that of the hot end temperature curve, that means the temperature difference between cold end and hot end plays a decisive role in thermoelectric output performance.

Under the actual flight heat flow, the size optimization achieved good results, the output performance increased by 64.8%, the concept of volume power density was introduced, the output power per unit volume increased by 119.6%, the space utilization was improved, and the structure volume and weight were reduced.

## Figures and Tables

**Table 1:** Properties of composite phase change materials.

Materials	Phase Change Temperature (K)	Phase Change Enthalpy (KJ/Kg)
**Paraffin**	328	200.1
**Paraffin with graphite foam**	324	152.7
**Erythritol**	391	337.6
**Erythritol with graphite foam**	390	261.1

**Table 2:** Optimization results.

	L_P (mm)	L_N (mm)	H1 (mm)	R (Ω)	P (w)	Efficiency (%)
**Basis**	1.5	1.5	2	0.25	**0.2617**	5.89
**Optimization**	1.519	1.687	1.003	0.217	**0.4313↑**	**4.99↓**

## References

[1] Zeleke M.A., Lai X., Liu L. (2020). A Peridynamic Computational Scheme for Thermoelectric Fields. Materials.

[2] Mirhosseini M., Rezania A., Iversen B., Rosendahl L. (2018). Energy Harvesting from a Thermoelectric Zinc Antimonide Thin Film under Steady and Unsteady Operating Conditions. Materials.

[3] Lim H., Jeong J.W. (2020). Numerical and Experimental Study on the Performance of Thermoelectric Radiant Panel for Space Heating. Materials.

[4] Nithyanandam K., Mahajan R.L. (2018). Evaluation of metal foam based thermoelectric generators for automobile waste heat recovery. Int. J. Heat Mass Transf..

[5] Chen W.-H., Wang C.-C., Hung C.-I., Yang C.-C., Juang R.-C. (2014). Modeling and simulation for the design of thermal-concentrated solar thermoelectric generator. Energy.

[6] Jia X.-D., Wang Y.-J., Gao Y.-W. (2017). Numerical simulation of thermoelectric performance of linear-shaped thermoelectric generators under transient heat supply. Energy.

[7] Liao X., Liu Y., Ren J., Guan L., Sang X., Wang B., Zhang H., Wang Q., Ma T. (2020). Investigation of a double-PCM-based thermoelectric energy-harvesting device using temperature fluctuations in an ambient environment. Energy.

[8] Wang W., Li X., Gu M., Xing Y., Bao Y. (2019). Low Temperature Joining and High Temperature Application of Segmented Half Heusler/Skutterudite Thermoelectric Joints. Materials.

[9] Wang P., Wang K.F., Wang B.L., Cui Y.J. (2019). Modeling of thermoelectric generators with effects of side surface heat convection and temperature dependence of material properties. Int. J. Heat Mass Transf..

[10] Liu Z.C., Zhu S.P., Ge Y., Shan F., Zeng L.P., Liu W. (2017). Geometry optimization of two-stage thermoelectric generators using simplified conjugate-gradient method. Appl. Energy.

[11] Demir M.E., Dincer I. (2017). Development and heat transfer analysis of a new heat recovery system with thermoelectric generator. Int. J. Heat Mass Transf..

[12] Chen W.-H., Huang S.-R., Wang X.-D., Wu P.-H., Lin Y.-L. (2017). Performance of a thermoelectric generator intensified by temperature oscillation. Energy.

[13] Fan S., Gao Y. (2018). Numerical simulation on thermoelectric and mechanical performance of annular thermoelectric generator. Energy.

[14] Zhang X., Tan G., Yang B. (2017). Impact Factors Analysis of the Hot Side Temperature of Thermoelectric Module. J. Electron. Mater..

[15] Meng Z.N., Zhang P. (2017). Experimental and numerical investigation of a tube-in-tank latent thermal energy storage unit using composite PCM. Appl. Energy.

[16] Chen M., He Y., Ye Q., Zhang Z., Hu Y. (2019). Solar thermal conversion and thermal energy storage of CuO/Paraffin phase change composites. Int. J. Heat Mass Transf..

[17] Khan Z., Khan Z., Ghafoor A. (2016). A review of performance enhancement of PCM based latent heat storage system within the context of materials, thermal stability and compatibility. Energy Convers. Manag..

[18] Miró L., Gasia J., Cabeza L.F. (2016). Thermal energy storage (TES) for industrial waste heat (IWH) recovery: A review. Appl. Energy.

[19] Sharif M.K.A., Al-Abidi A.A., Mat S., Sopian K., Ruslan M.H., Sulaiman M.Y., Rosli M.A.M. (2015). Review of the application of phase change material for heating and domestic hot water systems. Renew. Sustain. Energy Rev..

[20] Alva G., Lin Y., Liu L., Fang G. (2017). Synthesis, characterization and applications of microencapsulated phase change materials in thermal energy storage: A review. Energy Build..

[21] Yu J., Kong L., Wang H., Zhu H., Zhu Q., Su J. (2019). A Novel Structure for Heat Transfer Enhancement in Phase Change Composite: Rolled Graphene Film Embedded in Graphene Foam. ACS Appl. Energy Mater..

[22] Yu J., Kong L., Zhu Q., Zhu H., Wang H., Guan J., Yan Q. (2020). Thermal Stress Analysis of a Segmented Thermoelectric Generator under a Pulsed Heat Source. J. Electron. Mater..

[23] Kim H.S., Wang T., Liu W., Ren Z. (2016). Engineering Thermal Conductivity for Balancing Between Reliability and Performance of Bulk Thermoelectric Generators. Adv. Funct. Mater..

[24] Karri N.K., Mo C. (2018). Structural Reliability Evaluation of Thermoelectric Generator Modules: Influence of End Conditions, Leg Geometry, Metallization, and Processing Temperatures. J. Electron. Mater..

[25] Wang P., Wang B.L., Li J.E. (2019). Temperature and performance modeling of thermoelectric generators. Int. J. Heat Mass Transf..

[26] Yu J., Wang H., Kong L., Zhu H., Zhu Q., Li Q., Guan J. (2020). Analysis of temperature control effect of composite phase change structure used in thermoelectric conversion system. Appl. Therm. Eng..

[27] Caccia M., Narciso J. (2019). Key Parameters in the Manufacture of SiC-Based Composite Materials by Reactive Melt Infiltration. Materials.

[28] Asaadi S., Khalilarya S., Jafarmadar S. (2018). Numerical study on the thermal and electrical performance of an annular thermoelectric generator under pulsed heat power with different types of input functions. Energy Convers. Manag..

[29] Gong C.-L., Gou J.-J., Hu J.-X., Gao F. (2018). A novel TE-material based thermal protection structure and its performance evaluation for hypersonic flight vehicles. Aerosp. Sci. Technol..

[30] Wang B.L. (2017). A finite element computational scheme for transient and nonlinear coupling thermoelectric fields and the associated thermal stresses in thermoelectric materials. Appl. Therm. Eng..

